# Socioeconomic and demographic drivers of red and processed meat consumption: implications for health and environmental sustainability

**DOI:** 10.1017/S0029665116000100

**Published:** 2016-03-29

**Authors:** Angie Clonan, Katharine E. Roberts, Michelle Holdsworth

**Affiliations:** 1 School of Biosciences, University of Nottingham, Nottingham, UK; 2 ScHARR- School of Health and Related Sciences, University of Sheffield, Sheffield, S1 4DA, UK

**Keywords:** Red meat, Socioeconomic factors, Demographic factors, Environmental impact, Health

## Abstract

Red and processed meat (RPM) intake varies widely globally. In some high-income countries (HIC) the last decade has witnessed an overall decline or stabilisation in the consumption of RPM, in contrast to emerging economies where its consumption continues to increase with rising income and rapid urbanisation. The production and consumption of RPM have become major concerns regarding the environmental impacts of livestock in particular, but also because of associations between high RPM consumption and diet-related non-communicable disease. Therefore, it is important to identify socioeconomic and demographic drivers of the consumption of RPM. This paper explores how consumption of RPM differs with age, gender, socioeconomic status and in different global contexts. There are some key socioeconomic and demographic patterns in RPM consumption. Men tend to consume RPM more often and in higher quantities, and there is evidence of a social gradient in HIC, with lower socioeconomic groups consuming RPM more often and in larger quantities. Patterns for consumption with age are less clear cut. It is apparent that consumers in HIC are still consuming high levels of RPM, although the downward shifts in some socioeconomic and demographic groups is encouraging and suggests that strategies could be developed to engage those consumers identified as high RPM consumers. In low- and middle-income countries, RPM consumption is rising, especially in China and Brazil, and in urban areas. Ways of encouraging populations to maintain their traditional healthy eating patterns need to be found in low- and middle-income countries, which will have health, environmental and economic co-benefits.

Meat consumption garners polarising views in terms of its nutritional and environmental impact. Broadly speaking, the concerns fall into two groups: those associated with the production of meat consumed by the world's populations today (and projected increases) and those associated with the health consequences of meat consumption. The drivers of meat consumption are complex and influenced by an inter-related system of factors including culture^(^^1^^,^^2^^)^, taste^(^^3^^)^, cost^(^^4^^)^, religion^(^^2^^,^^5^^)^, gender and socioeconomic status (SES)^(^^6^^)^.

## Health consequences of red and processed meat consumption

Concerns associated with the health consequences of red and processed meat (RPM) consumption focus in particular on the emerging literature on their health effects on some cancers^(^^7^^,^^8^^)^, CVD^(^^9^^,^^10^^)^, obesity^(^^11^^,^^12^^)^, type 2 diabetes^(^^13^^)^ and antibiotic resistance^(^^14^^)^. Some of these negative health consequences depend on the type of meat. Processed meat includes meat products that have been modified to change the taste or extend shelf life through curing, smoking, salting or adding preservatives. Frequently consumed examples are shown in Table 1. The consumption of processed meats has been associated with all-cause mortality^(^^15^^)^, which may partially result from the higher saturated fats and cholesterol contained in the processed meats, but is most likely due to the processing itself, i.e. salting, curing or smoking. Whilst lengthening shelf life or improving flavour, processed meats also contain known carcinogenic precursors such as polycyclic aromatic hydrocarbons, heterocyclic aromatic amines and nitrosamines^(^^15^^)^ and they are high in salt^(^^16^^)^. This may shed light on recent research suggesting that processed meat consumption increases risk of cancer, as eating 50 g of processed meat a day increases the chance of developing colorectal cancer by 18 %^(^^8^^)^. Indeed large cohort studies and meta-analyses indicate that a high consumption of processed meat is associated with increased overall mortality, but unprocessed meat is not^(^^16^^)^.

**Table 1.** Types of red and processed meats

Type	Description	Food examples
Red meat	Meat from mammals which is higher in myoglobin than a white meat.	Lamb, mutton, beef, pork, veal, goat, horse
Processed meats	Meat products that have been modified to change the taste or extend shelf life through curing (adding salt enriched with nitrates and nitrites), smoking, salting or adding preservatives. Most contain some beef or pork, but may also contain poultry, offal, other red meats or meat by products	Ham, sausages, salami, bacon, hot dogs, corned beef, beef jerky, ham, canned meat and meat-based sauces

On the other hand, evidence that lean red meats (Table 1) *per se* are carcinogenic is limited. It is still widely acknowledged that lean red meat is an important complete protein source, in addition to contributing to essential micronutrient requirements, particularly iron, zinc and B vitamins^(^^17^^)^. Iron deficiency is the most prevalent micronutrient deficiency in the world, affecting over 1 billion people and if untreated, it can lead to anaemia, with adolescent girls and women of reproductive age being particularly at risk^(^^17^^)^. Balancing these environmental and health tensions is a challenge for the public's health.

This complexity make it particular difficult for consumers to determine whether or not to include RPM in their diets, and if so, how much to include^(^^18^^)^.

## Environmental sustainability and meat consumption

The environmental sustainability of meat consumption has become a concern globally for several reasons including resource inputs^(^^19^^,^^20^^)^, planetary limits^(^^20^^–^^23^^)^, environmental degradation^(^^24^^–^^26^^)^ and animal welfare^(^^6^^)^. The agri-food sector accounts for over 30 % of greenhouse gas emissions (GHGE) globally and the livestock sector alone contributes 15 % of GHGE^(^^23^^)^. Ruminant meats (beef and lamb), for example have GHGE per g of protein that are 250 times greater than legumes^(^^22^^)^. It has been estimated that halving meat, dairy and egg consumption in the European Union would reduce GHGE by up to 40 % and reduce cropland use for food production by almost a quarter^(^^27^^)^. Beef requires much more irrigation water per kcal eaten compared with other protein sources. However, the environmental impact of red meat depends on the way it is produced, for example, if ruminant animals are grazed on land unsuitable for crops and fed crop residues, then dairy and meat production can provide environmental benefits through nutrient recycling^(^^22^^)^.

Comparisons between vegetarian and meat-based diets have illustrated vast differences in their environmental impact, with a meat-based diet using almost three times more water, thirteen times more fertiliser, and 1·4 times more pesticides than a meat-free diet^(^^28^^)^. Animal-based foods also generate more GHGE than do plant-based foods, with the exception of fruit and vegetables grown in greenhouses^(^^29^^)^. Food production is the largest contributor of GHGE in the agri-food system and its inefficiency is of concern, i.e. intensive livestock farming uses the equivalent of 37·656 kJ (9 kcal) of grain to make 4184 kJ (1 kcal) of beef, a proportion that becomes 4/1 for pork and 2/1 for chicken^(^^30^^)^. Hence, the future sustainability of meat remains one of the biggest challenges for a sustainable agri-food system.

## Trends in red and processed meat consumption globally

In spite of health and environmental concerns, red meat consumption continues to rise in some parts of the world, as part of the global transition to a diet high in fat and sugar, increasing meat consumption and decreasing fruit, vegetables and cereals^(^^31^^,^^32^^)^, particularly in urban areas due to changing dietary habits related to rapid urbanisation. Overall, processed meat intakes have been stable over time on a global level (1990–2010), whereas red meat intake has increased, based on data synthesised from 113 countries from food balance sheets and food consumption surveys^(^^33^^)^. Only in East Asia has unprocessed red meat intake significantly increased during this period. Country-specific intake varies enormously for both red meats (3·0–124·2 g/d) and processed meats (2·5–66·1 g/d)^(^^33^^)^.

In higher income countries such as the UK, consumption remains high, although there have been shifts in the type of meat consumed^(^^34^^)^. Poultry consumption has increased 5-fold since the 1960s, probably due to a reduction in the relative price of chicken, whereas consumption of beef and lamb have declined over the same period^(^^34^^)^. As low- and middle-income countries grow economically, the consumption of meat increases with available income, leading to vast disparities in intake between high, middle and lower income populations between and within countries^(^^31^^)^.

**Fig. 1.**
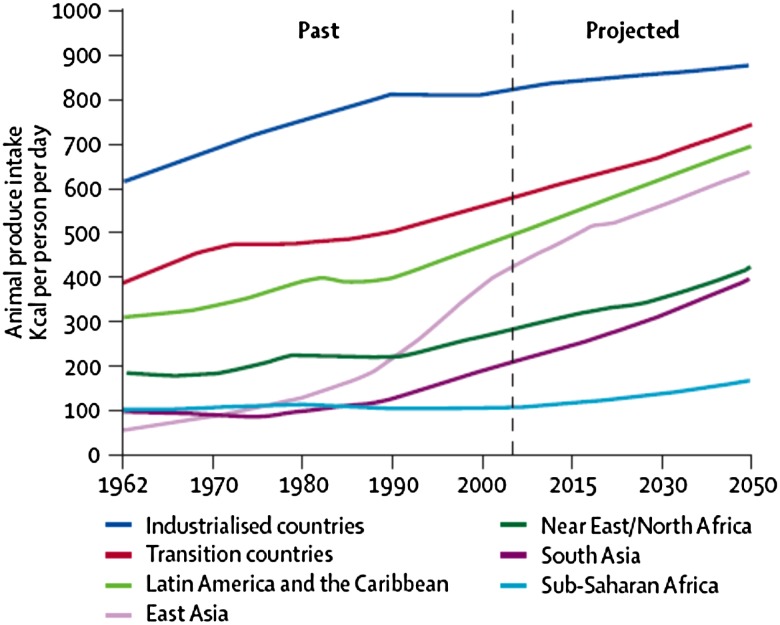
Global consumption trends of animal produce. Source: McMichael *et al*., Lancet, using data from the Food and Agricultural Organisation.

The average meat consumption globally is 100 g/d per person, but this average figure masks the huge diversity of intakes, particularly between countries. For example, in low-income countries the average daily meat consumption is half the global average, whilst it is double that in high-income countries (HIC)^(^^20^^)^. Of great concern, is that meat consumption is rising (Fig. 1), especially in emerging economies where consumption was previously low, such as those in South and East Asia. As the global price of meat has decreased it has become more accessible in low- and middle-income countries, especially for processed meats of poor quality.

Globally, the USA has the highest consumption. In France, meat consumption has been falling since 2000. Meat consumption in rapidly emerging economies such as Brazil and China has increased over the last 30 years, with intakes doubling and tripling, respectively^(^^2^^)^.On the African continent, only South Africans have intakes similar to that seen in China. Other sub-Saharan African countries remain low consumers of red meat. Since the global population is expected to rise to 9·6 billion by 2050^(^^35^^)^, it is predicted that demand for meat and animal products will continue to rise, causing further environmental concerns.

## Sociodemographic patterns in red and processed meat consumption

### Gender differences in red and processed meat consumption

Several studies have reported than men consume more RPM than women. For example, data from the UK's most recent National Diet and Nutrition Survey (NDNS) collected between 2008 and 2011 highlight differences in consumption of RPM for gender, age and SES^(^^36^^)^. In this analysis, men consumed significantly higher (*P* < 0·05) quantities of RPM (both total g and g/4184 kJ (1000 kcal) consumed) as Table 2 illustrates.

**Table 2.** Red and processed meat consumption by gender (data from UK National Diet and Nutrition Survey, 2008–2011; *n* 1959)

	Males	Females
Red meat per 4184 kJ (1000 kcal)	45·32*	38·38
Total red meat (g)	86·89*	56·76
Processed meat per 4184 kJ (1000 kcal)	10·97*	9·49
Total processed meat (g)	21·59*	14·00

*P < 0·05.

This is supported by analysis conducted by Maguire and Monsivais^(^^37^^)^, who also found that men consume more RPM than women by analysing 3 years of the UK's NDNS data, based on a combined RPM variable^(^^37^^)^. Research conducted in Nottinghamshire, UK of 842 participants also illustrated differences in meat consumption by gender; as women were significantly more likely (*P* < 0·01) to consume ⩽1 portion of RPM per d, compared with men. No other significant relationships in terms of consumption were observed in this study for age or SES, despite more positive attitudes towards consuming less meat and animal welfare by older respondents^(^^6^^)^. Literature examined from other HIC within Europe, for example in Germany, additionally indicates that men consume more RPM than women^(^^38^^)^. Further afield, the US National Health and Nutrition Examination Surveys data also illustrate that men consume more of every type of meat than women (*P* < 0·0005) and highlights an on-going trend of women reducing their consumption of red meat^(^^39^^)^.

These differences in reported consumption could derive from previously highlighted differences in attitudes towards eating meat between men and women, possibly connected to greater motivation regarding personal health or animal welfare concerns of women^(^^6^^)^. The sociological literature highlights a link between perceived ‘virulent masculinity’ and meat consumption^(^^39^^)^ and this, combined with the use by some fast food retailers of gender-based advertising strategies, which specifically target male consumers, could contribute to greater consumption and possible over reporting of meat consumption amongst some men. Of further note in the literature is the link between vegetarianism and feminism^(^^40^^)^, which can be summarised by a strong sense of ethical consideration towards animals, and is enacted through ‘cruelty free consumption’ by abstinence of animal products in the diet^(^^41^^)^. These discourses would benefit from further exploration in order to better understand the relationships which exist between gender and meat consumption, and to determine whether links exist between red and/or processed meat in particular.

### Age differences in red and processed meat consumption

Analysis of UK nationally representative NDNS data showed no significant differences in consumption of red or processed meat between age groups (determined by one-way ANOVA)^(^^36^^)^. However, a statistically significant difference between age groups was observed for total red meat per 4184 kJ (1000 kcal) of food energy intake (*F* (3, 2030) = 2·825, *P* = 0·37). A Tukey's *post hoc* test revealed that those aged 46–60 years consumed significantly more red meat (43·96 ± 29·84, *P* = 0·41) compared with younger adults aged 19–30 years (38·20, ±27·48). This higher consumption in middle age may fall again with further ageing, as illustrated in a recent report which stressed that people over the age of 65 years eat less RPM than younger respondents in the UK^(^^34^^)^, a finding which is supported by a longitudinal British cohort study evidencing a reduction in meat consumption as people age^(^^42^^)^, which concurred with previous research^(^^6^^)^; however, young people were also more likely to report that they do not eat any meat at all^(^^34^^)^. Similar contradictions in age related to RPM consumption were highlighted by Wang *et al.*^(^^39^^)^, when analysing several US datasets, in that the National Health and Nutrition Examination Surveys data showed that meat consumption decreased with age, whereas the more recent ‘Continuing Survey of Food Intakes by Individuals’ dataset evidenced older groups consuming more meat.

Differing attitudes held by older adults towards the source of their meat and animal welfare have been highlighted in previous research, which has also noted that those of middle age and above (>46 years) were more likely to frequently purchase meat considered ‘sustainable‘^(^^6^^)^. This may account for some of the reported consumption differences, as older adults in the UK may remember the experience of food rationing during the Second World War^(^^6^^)^. Deteriorating dentition and a decline in chewing capacity may also play a role in older adults consuming less meat, in particular red meat which is often tougher to chew than poultry.

### Socio-economic status differences in red and processed meat consumption

**Fig. 2.**
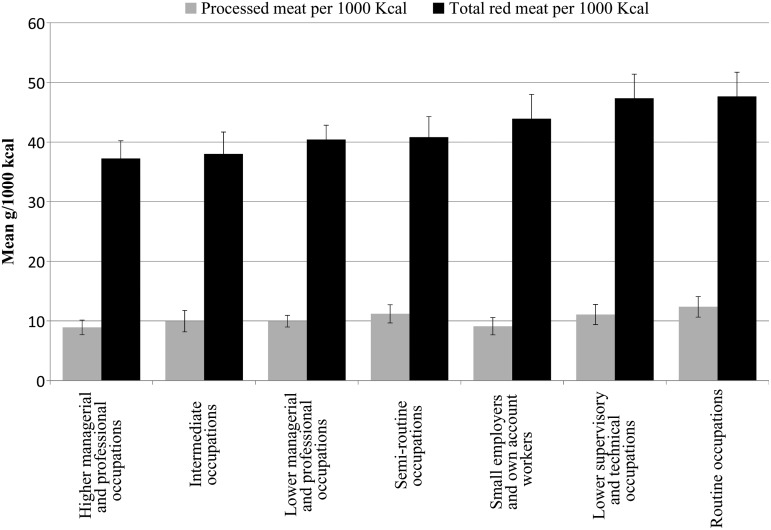
Mean processed meat and total meat consumed (g per 4184 kJ (1000 kcal)) in the UK by occupational group (data from UK National Diet and Nutrition Survey, 2008–2011; *n* 1959).

The relationship between SES (education, income, occupation) and RPM consumption in high income settings suggests that higher intakes are evident in low SES groups, although the distinction between RPM is not clear cut. In the UK, NDNS data indicate a statistically significant difference in RPM consumption by SES determined between occupational groups for total red meat (*F* (7, 1993) = 3·93, *P* < 0·001), processed meat (*F* (7, 1993) = 2·78, *P* = 0·007), total red meat per 4184 kJ (1000 kcal) (*F* (7, 1993) = 4·56, *P* < 0·001) and processed meat per 4184 kJ (1000 kcal) (*F* (7, 1993) = 3·28, *P* = 0·002). A Tukey's *post hoc* test revealed patterns that indicate a socioeconomic gradient in consumption of RPM, which was particularly notable by occupational group, as shown in Fig. 2. Those in higher managerial and professional occupations reported consuming significantly less red meat per 4184 kJ (1000 kcal) (37·24 g, ±26·32) than those in lower supervisory and technical occupations (47·35 g ±29·06), *P* = 0·004 and those in routine occupations (47·65 g ±31·31), *P* = 0·001. Similarly, those in lower managerial and professional occupations and intermediate occupations reported consuming significantly less red meat per 4184 kJ (1000 kcal) (40·41 g, ±28·5; 38·02 g, ±25·52, respectively) than those in routine occupations (47·65 g, ±31·31), *P* = 0·038 and *P* = 0·019, respectively. Those in higher managerial and professional occupations also reported consuming significantly less processed red meat per 4184 kJ (1000 kcal) (8·91 g, ±10·84) than routine occupations (12·37 g, ±13·30), *P* = 0·25. Those in lower supervisory and technical occupations and those in routine occupations reported consuming significantly more processed red meat (19·12 g, ±22·2; 20·98, ±25·88, respectively) than those who have never worked (7·90 g, ±12·20), *P* = 0·048 and *P* = 0·008, respectively.

The social gradient highlighted is an important outcome of this analysis, because of the implications for public health. Maguire and Monsivais^(^^37^^)^ also present evidence of a social gradient in intake, with a significant trend across each SES indicator; for example the lowest earning households consumed 15·7 g/d more RPM than the highest earning households, those with no formal qualifications consumed 21·9 g/d more RPM than those with a degree qualification, and higher managerial and professional occupations consumed 25·5 g/d less RPM than those in routine occupations. A study in France^(^^43^^)^ also found a positive relationship between low education level and lower meat intake.

People in higher socioeconomic groups may have a greater awareness of the health implications associated with over consumption of RPM, which could also lead to an increased consumption of other more beneficial food groups, for example oily fish or fruits and vegetables. In the case of fish, although it is a healthier choice, viewing it as an alternative protein source to meat carries serious implications in terms of supply, as stocks cannot meet current recommendations^(^^44^^)^. Levels of awareness and attitudes towards animal welfare have been shown in Dutch consumers to influence meat purchasing behaviour in terms of choosing meat which is ‘organic’ and ‘free range‘^(^^45^^)^, and interestingly a relationship with SES exists between those abstaining from meat, as research suggests there is a higher level of education amongst those choosing to be vegetarian^(^^46^^)^ and higher meat intake in people with lower SES.^(^^47^^)^

This concurs with the findings of a study^(^^2^^)^ synthesising panel data for 120 countries over a long period (1970–2007) which analysed the link between income and meat consumption. The study reported that meat consumption is higher initially at higher income levels but then over time, higher levels of income are associated with lower levels of meat consumption, leading to an inverted U-shaped curve of consumption. This may be explained in part by Bourdieu's theory of distinction^(^^48^^)^ and the ways in which people make decisions about their meat intake may be reflective of their social standing in society. It could be that when meat is initially an expensive and inaccessible food it is appealing to those in higher SES groups so they can distinguish themselves from the ‘masses’. As RPM become more accessible to the wider population it then loses its appeal as it is no longer associated with the ‘taste of luxury’.

One powerful determinant of choice in food is cost, and this is likely to play a role in driving processed meat intake, as it is often cheaper than lean ‘carcass’ meat which will not have had additional substances added to enhance flavour, increase shelf life or indeed add value for the producer, as is the case with many processed types of meat. Cost has also been shown to be a factor inhibiting economically disadvantaged groups from accessing health and sustainable diets in other research^(^^4^^)^. Lower food prices have been linked to greater consumption of red meat globally^(^^3^^)^. Altruistic motivations are likely to have an influence on consumers from higher socioeconomic groups consuming less RPM, for example the environmental footprint associated with livestock production^(^^26^^)^ or animal welfare concerns.

## Challenges in synthesising red and processed meat consumption data

Despite advances in food consumption and nutrition surveillance research, the ability to identify trends and associations from the available primary data remains challenging, for several reasons. Firstly, the need to decide whether to explore food supply data, such as those datasets provided by the Food and Agricultural organisation of the United Nations, which indicate quantities of particular foodstuffs available in specific countries, or to focus on data from national dietary surveys. Some studies have utilised both types of data^(^^49^^)^, but this can make comparisons problematic, particularly when food wastage is estimated to be one-third for HIC such as the UK^(^^50^^)^. Therefore the NDNS survey, which assesses consumption, provides a more accurate picture; however, as with all self-reported food consumption data, potential under and over reporting is acknowledged^(^^51^^,^^52^^)^.

Additionally, there is no clearly agreed definition as to what constitutes ‘processed meat’, although we have provided a summary in Table 1 of this. The US National Health and Nutrition Examination Surveys currently places cured meat such as bacon or ham within the ‘fresh meat’ category, unlike the UK and WHO which considers cured meats such as bacon and ham to be ‘processed meat’. Many studies to date have conducted analyses by considering both red carcass and processed red and white meat as a single variable^(^^37^^,^^49^^)^, despite the very different health outcomes associated with the consumption of processed meat which are now emerging from the literature^(^^15^^)^. Therefore improving data collection methods and an official agreed definition for what constitutes ‘processed meat’ are essential for the future understanding of diet and disease associations.

## Conclusion

An unprecedented shift in RPM consumption of most individuals in HIC is required to reduce its environmental and health impacts. There are some key socioeconomic and demographic patterns in RPM consumption, which can be useful to guide interventions, for example men tend to consume higher quantities, and the clear social gradient presented with lower SES groups consuming larger quantities in HIC. Patterns for consumption with age are less clear cut. It is apparent that consumers in HIC are still consuming high levels of RPM, although the downward shifts in some socioeconomic and demographic groups is encouraging and suggests that strategies could be developed to engage individuals identified as high RPM consumers, in particular young males and those from lower socioeconomic groups. In low- and middle-income countries, RPM consumption is rising, especially in China and Brazil, and in urban areas. Ways of encouraging populations to maintain their traditional eating patterns need to be found and will have health, environmental and economic co-benefits.

Meat is a heterogeneous commodity in terms of its nutritional value, as processed meats have the most negative health value, whereas lean red meat is an important source of protein and micronutrients. Dietary patterns characterised by high RPM consumption tend to be lower in plant-based foods, for example fruits and vegetables^(^^49^^)^. The promotion of plant-based diets, including protein alternatives (such as beans, pulses, nuts, etc.) should be encouraged, as this would have the advantage of enhancing the healthiness of diets and reducing the environmental consequences of the agri-food system.

Reductions in RPM consumption is unlikely to happen without major policy shifts to support individuals in making the necessary changes. Any policy solutions need to account for the multitude of nutritional problems that co-exist in different contexts and the need to provide supportive environments. Social media campaigns may help to engage a wider audience in some contexts. Similarly, macro level approaches that have a more direct influence on purchasing decisions, for example financial incentives, and cost could be modelled to ascertain which particular RPM products have higher externalised costs to both the environment and public health. Human health is a stronger motivation to reduce RPM than environmental sustainability^(^^6^^)^. A first step will be for nutritionists and health professionals to raise public awareness about the link between eating RPM on both health and environmental sustainability, to build support for further action.
